# Effect of D-Panthenol on Corneal Epithelial Healing after Surface Laser Ablation

**DOI:** 10.1155/2018/6537413

**Published:** 2018-11-12

**Authors:** Islam Mahmoud Hamdi

**Affiliations:** ^1^Associate Professor, Ophthalmology Department, Faculty of Medicine, Ain Shams University, Cairo, Egypt; ^2^Ophthalmology Consultant, The Eye Consultants Center, Jeddah, Saudi Arabia

## Abstract

**Purpose:**

To study the effect of D-panthenol (provitamin B5) on corneal epithelial healing, in cases of surface laser ablation. *Patients and Methods*: 45 eyes, of 45 patients undergoing laser surface ablation, received D-panthenol 2% in one eye and artificial tear drops of similar composition not containing D-panthenol in the other eye, postoperatively, for 2 months. Patients were examined daily for 3 days after the procedure. They were then examined weekly for 1 month. An additional examination was made after 2 months. Visual acuity (Log MAR) was assessed at every visit. Rate of healing (% of covered area) and subjective sensation of discomfort (scale 0–5) were assessed in the 1st 3 visits. Residual manifest cylinder (D) (as a parameter of corneal irregularity) and corneal clarity (epithelial and stromal haze) were assessed from week 1 to month 2.

**Results:**

During the first 3 days, both groups showed statistically nonsignificant (*P* > 0.05) results. From week 1 to month 2, eyes receiving D-panthenol showed better vision and less residual cylinder (*P* < 0.05) at week 1. For all other parameters, and at different examinations, both groups showed a statistically nonsignificant (*P* > 0.05) difference. Still, eyes receiving D-panthenol showed better values at the majority of the parameters tested.

**Conclusion:**

D-Panthenol effect on corneal epithelial regeneration is of minimal clinical relevance. A different dosage and a larger sample of patients might reveal a statistical relevance. This trial is registered with https://doi.org/10.1186/ISRCTN81441126.

## 1. Introduction

The integrity of corneal epithelium is essential for various corneal functions, including but not limited to clarity and immunity [1]. The response to a defect in the corneal epithelium has long been studied [2–7]. The most accepted theory that explains that response is “*X*, *Y*, and *Z* theory” [8], where it suggests the replication of stem cells, then a horizontal migration to fill up the defect, and finally a vertical growth in order to end up with a matured, five-layered, stratified squamous, nonkeratinized epithelium.

Different factors, both internal and external, may contribute, positively or negatively, to this process [9–16]. While agents like corticosteroids [9] and antimetabolites [10] are well known to hinder epithelial regeneration; others like autologous serum [11], umbilical cord serum [12], and various growth factors [13] were found to promote it.

D-Panthenol, the precursor of vitamin B5, possesses an established positive effect on epithelium healing in general [17, 18]. It acts through moisturizing surfaces and creating a barrier effect [17]. Still, at the molecular level, its mechanism is not yet established [17].

Recently, ophthalmic preparations have included D-panthenol in its ingredients, in order to get benefit from its healing effect, at the corneal level [19–21]. Yet, few studies have tackled this issue [19–24].

In this study, we assessed the effect of D-panthenol 2% ophthalmic preparation (Augé Vitamin™, Köln, Germany) on corneal epithelial healing, when the defect is induced during a surface laser ablation, intended for vision correction.

## 2. Materials and Methods

This is a prospective study which included 45 patients. Each patient underwent a surface ablation procedure in both eyes. One eye received D-panthenol (provitamin B5) in propyl-methyl cellulose (Augé Vitamin™), and the other eye received artificial tear drops in the form of carboxy-methyl cellulose (Refresh™- Allergan- Irvine-California). For purpose of randomization, the first 23 patients received Augé Vitamin™ in the right eye and Refresh™ in the left. The second 22 patients received the opposite. Eyes with Augé Vitamin™ were considered as cases (group A), and those who received Refresh™ served as control group (group B).

### 2.1. Settings

The study was conducted in the Eye Consultants Center, Jeddah, Saudi Arabia.

### 2.2. Recruitment

Patients were enrolled into the study if they were suffering from ametropia and seeking vision correction. Age should be within 18–45 years at time of surgery. BSCVA should reach at least 20/20 in each eye. Eyes should be otherwise free from any pathology in terms of ocular surface (dry eye, inflammation, corneal scar, vascularization, etc.), anterior segment (cataract, glaucoma, uveitis, etc.), and posterior segment ones. Eyes should also possess corneas not susceptible for postoperative ectasia.

Cases were examined for visual acuity with Snellen's chart. Refraction was performed as manifest and cycloplegic. Anterior segment was examined by slit lamp and posterior segment by dilated fundoscopy. Cases were examined for eligibility for laser vision correction by corneal tomography (Pentacam™, Oculus, Germany) to detect the susceptibility for postoperative corneal ectasia.

### 2.3. Surgical Procedure

After prepping and draping, the conjunctival sac was washed with ample amount of isotonic saline. Corneal epithelium was mechanically debrided after installation of 20% ethyl alcohol in an 8.0 mm well for 20 seconds. Laser ablation was performed using Wavelight® EX-500 platform (Alcon laboratories, Inc.). Ablation was centered on the pupil, and axis of astigmatism was respected by a pupil tracker and iris and limbus registration system.

Ablation depth was determined according to error of refraction, optical zone (OZ), and the profile of ablation. In myopic eyes, refraction was adjusted according to a nomogram, adding 0.25 D for each −3.0 D < −3.0 D of total refraction (sphere + cylinder), an extra 0.25 D for individuals >35 years, to compensate for hyperopic shift. Hyperopic eyes were treated without modification of manifest refraction.

Standard optical zone was 6.5 mm. 7.0 mm was chosen for hyperopic cases, and 6.0 mm was chosen for cases <500 *µ*m initial corneal thickness. A standard ablation profile (Custom Q™ Wavelight® EX-500 platform, Alcon laboratories, Inc.) was utilized for all cases.

After ablation, a cellulose sponge soaked with 0.02% MMC was applied for 1 second for each 2 *µ*m ablated, with a minimum of 20 sec, to avoid postoperative stromal haze. Residual MMC was washed with 20 ml isotonic saline. A bandage contact lens was applied. A fixed combination antibiotics (oxyfloxacin) and corticosteroids (0.1% prednisolone) (Loxtra™, Jamjoum Pharma, Saudi Arabia) was installed to the conjunctival sac. Patients were discharged after being informed for safety instruction in the early postoperative period.

All surgeries were performed by same surgeon (IH).

### 2.4. First Postoperative Stage: Stage of Epithelial Defect

In addition to the tested agents mentioned above (Augé Vitamin™ and Refresh™), patients received only antibiotic eye drops, Gatifloxacin (Zymaxid™, Allergan, Irvine, CA). All drops were prescribed Qid. To avoid corneal infection, patients were instructed to remain indoors during the epithelial defect stage (except for examination visits). They were also instructed to avoid touching the eye, washing face, and exposure to pollution and smoke.

Patients were received for examination daily, for 3 days, until complete epithelial defect closure. During these visits, UCVA was tested, percentage of epithelial closure was estimated, and assessment of patient comfort was done, for each eye separately.

UCVA was measured in presence of −0.25 D bandage contact lens, assessed in 20-feet scale and then converted into Log MAR scale for statistical purposes.

Percentage of epithelial defect healing was assessed by the same examiner (IH).

Subjective assessment for comfort was evaluated by asking the patient to evaluate pain and itching in each eye, giving the score of 0 for no discomfort at all and 5 to severe intolerable symptoms.

### 2.5. Second Postoperative Stage: Stage of Epithelial and Stromal Remodeling

After complete epithelial defect closure, bandage contact lens was removed. Patients were instructed to resume normal life activities except exposure to UV rays in form of sun light for 4 months.

Antibiotic drops were discontinued and replaced by topical corticosteroids, rimexolone 1% (Vexol™, Alcon, Fort Worth, Texas) Qid for 2 months. Tested agents were continued for 2 months.

Patients were received for examination at 1, 2, and 3 weeks and 1 and 2 months from the surgery day.

They were tested for UCVA similar to the first postoperative stage.

Residual subjective refraction was measured, and residual cylinder was considered as a sign for corneal regularity.

Corneal haze was evaluated in terms of presence and level (epithelial or stromal).

All patients signed an informed consent, explaining in details the procedure and the nature of the study. An approval was received from the ethical committee in the center prior to initiation of the study. The study respected the tenets of Declaration of Helsinki.

Statistical analyses were performed using SPSS software, version 12 (SPSS Inc., Chicago, Illinois). Descriptive analysis was performed by calculating mean±standard deviation and range for quantitative data. For qualitative data, frequencies were represented by a number and percentage. For parametric values, a between-group comparison was performed with Student's *t*-test for quantitative data and with chi-squared [2] test for qualitative data. For nonparametric values, the Mann–Whitney *U*-test was used for between-group comparison. *P* < 0.05 was considered statistically significant.

## 3. Results

Among the 45 patients included in this study, 37 (82.2%) were females and 8 (17.7%) were males. Mean age of patients was 29.3 ± 6.9(21–43) years.

Group A and B were matched in terms of preoperative refraction, ablation zone size, and depth of ablation.  Table 1 summarizes preoperative data.

**Figure 1:**
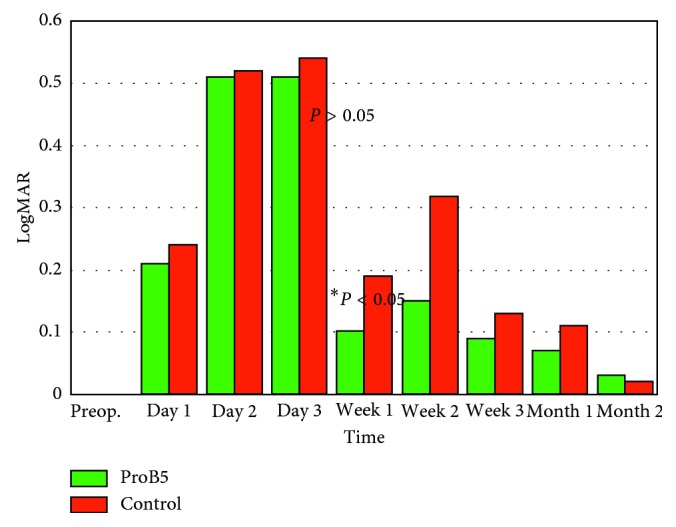
Uncorrected visual acuity (log MAR), with statically nonsignificant difference (*P* > 0.05) between both groups except for values at 1st week (*P* < 0.05).

**Figure 2:**
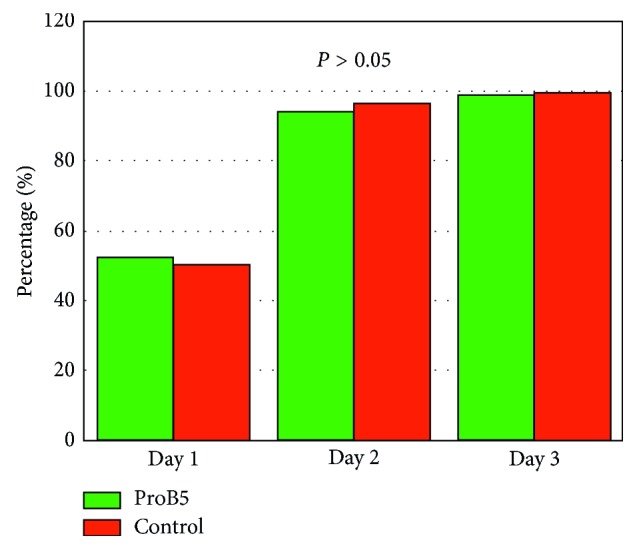
Rate of epithelial defect healing (%) with statically nonsignificant difference (*P* > 0.05) between both groups.

At the first postoperative stage (epithelial defect), both groups showed *nonsignificant statistical difference* (*P* > 0.05) in terms of visual acuity (Figure 1), the rate of healing (Figure 2), and the feeling of discomfort during epithelial defect. Still, the D-panthenol group showed better values in visual acuity throughout the whole period. A similar result was found in the rate of healing and discomfort score on the first day.  Table 2 presents the results at this stage.

**Figure 3:**
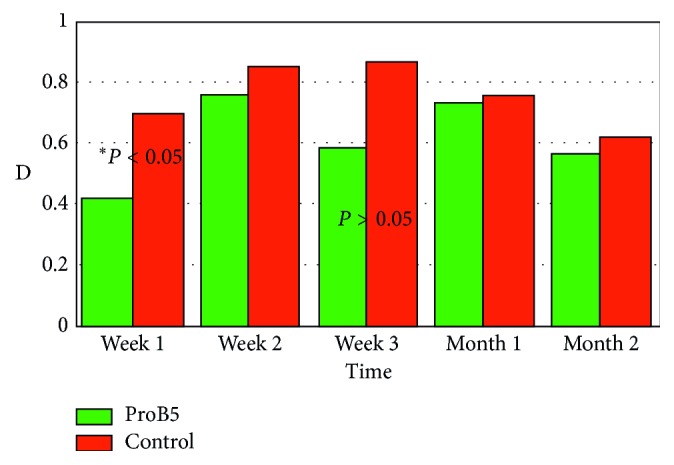
Residual cylinder (D), with statically nonsignificant difference (*P* > 0.05) between both groups except for values at 1st week (*P* < 0.05).

At the second postoperative stage (epithelial and stromal remodeling), both groups showed similar results, except for a *statistically significant difference* (*P* < 0.05), in favor of the D-panthenol group, in terms of visual acuity (Figure 1) and residual cylinder (Figure 3) at the first week. Otherwise, all results were *statistically nonsignificant* (*P* > 0.05), in terms of visual acuity, residual cylinder, and haze, at every point of examination. However, D-panthenol group always presented with values better than control in terms of residual cylinder. This applies also for visual acuity, with the exception of last visit, at 2 months.

**Figure 4:**
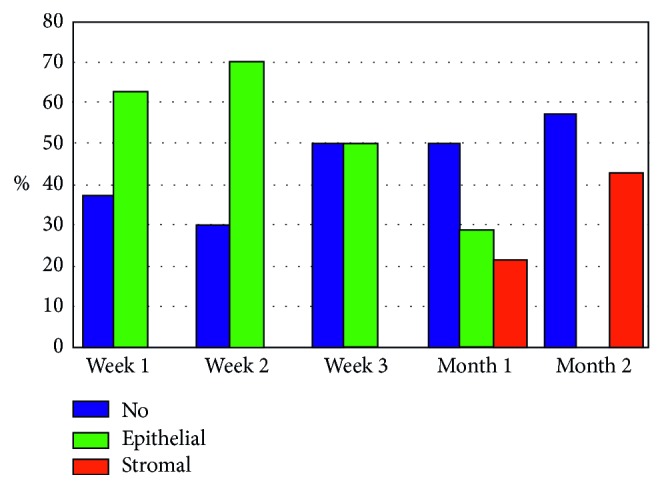
Haze in D-panthenol group.

**Figure 5:**
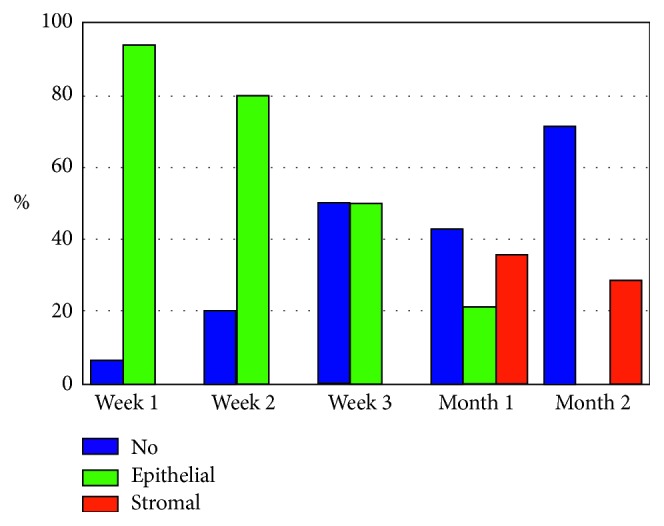
Haze in control group.

The D-Panthenol group showed rapid initial clarity of epithelial haze (1 and 2 weeks) and a nearly similar pattern of clarity with control, hence forward (Figures 4 and 5).  Table 3 presents all values at this stage.

## 4. Discussion

D-Panthenol proved to be of significant value in epithelial regeneration [17, 18]. This encouraged pharmaceutical companies to incorporate it in ophthalmic preparations [19–21]. In order to assess its role, it was of great relevance to standardize all factors in comparing its effect versus a control. It was also empirical to provide a condition that gives enough time and data to build up an objective judgment. For this reason, in this study, epithelial defect, created iatrogenically for laser vision correction, was chosen. This gives a controlled epithelial defect, large enough to produce a full healing procedure. At the same time, fellow eyes were completely under the same influences (Table 1) and only deprived from the D-panthenol effect. The duration (2 months), however, was enough to reveal most of the expected changes, but may have been short to express extended reactions like delayed haze.

In view of the *X*, *Y*, and *Z* theory [8], the first 3 days, after epithelial defect creation, correspond to the *X*-*Y* phase. This was observed for the speed of surface area coverage, the effect of vision, and subjective patient perception of the treatment (in terms of feeling of discomfort).

The first item that captures interest is speed of epithelial defect coverage. This would be the most anticipated point and visible for the examiner at the same time. At this point, D-panthenol showed no difference (Table 2; Figure 2). Nevertheless, it was noted that nearly all eyes, at both groups, have completed epithelial defect closure in 3 days. This might be attributed to the artificial tears effect (methyl cellulose), rather than the added pro-vitamin B5.

Similar to healing rate, both groups showed similar discomfort scores, during epithelial defect (Table 2). This is expected to be a consequence of the bare nerve endings, which in this situation, would not differ.

During epithelial defect closure, visual acuity showed no statistically significant difference (*P* > 0.05) (Table 2); yet, the eyes receiving D-panthenol showed values better than fellow eyes.

After complete epithelial defect closure, ocular surface still needs few weeks to normalize and “smoothen up.” This corresponds to the *Y*-*Z* phase. Observing corneal changes at this stage gives an idea about the prolonged effects on factors related to epithelial healing. For this reason, treatment and observation extended up to 2 months.

During the second stage, D-panthenol group showed superior values, in term of vision, which were only significant at the 1st week (Table 3; Figure 1).

The results of visual acuity in the 2nd phase could be explained by the measurement of the residual manifest cylinder. During this stage, a residual cylinder will result from the surface irregularity, which depends on the progress of healing. It does not represent a direct outcome of laser ablation and is in constant change in the first few months, after the procedure. In this case, it showed exactly the same outcome of vision, which is superior levels for D-panthenol all through the study, but only significant at the first week (Table 3, Figure 3).

The last point of comparison was corneal clarity, after complete epithelial defect closure. We referred to these points during observation as “haze.” It was observed at 2 levels: epithelium and stroma.

When fully healed and regularized, corneal epithelium is invisible. On the contrary, stromal haze is a well-known clinical situation after surface laser ablation [25–28]. It depends on various factors, but collectively, on rapid and smooth epithelial healing. At this point, we referred only to the presence of haze and not on its grade, at either level. In terms of clarity, both groups showed statistically nonsignificant difference (Table 3). However, eyes receiving D-panthenol showed initial clearer corneas (Figures 4 and 5), which coincides with the results of residual cylinder and vision outcome.

In an attempt to review the literature on the effects of D-panthenol on the eye and compare previous results to the current study, a few references were retrieved [19–24]. Some of them were published in non-English language [19, 20, 23]. Some of them utilized lab prepared solutions rather than commercially available medications, which might affect the standardization of the outcome [22–24]. While in this study, the tested agent was in drop form, other studies used rather gel and ointment forms [19–21].

This factor may affect retention time and hence clinical effect. Similarly, concentration differed in some studies (5%) [19–22]. Among the retrieved researches, only one was of comparative nature [22], where Baumeister et al. found similar results to ours: eyes treated with D-panthenol showed superiority over placebo but which was not statistically strong enough. In spite of the effort to bring the epithelial defect, subject of study, to the most objective conditions, the study could be limited due to the nature of defect itself, being a result of alcohol application and exposure to Excimer laser and mitomycin-C. It is not confirmed whether these elements might interfere with the D-panthenol action or not. Another limitation is the absence of a dose-titrated medication. Comparison was made between the commercially available concentration (2%) and its absence only. A suggested work is to vary the D-panthenol concentration and observe the differences in outcome.

In spite of some clinical benefits, observed during the course of the study, a statistical benefit could not be revealed completely. So, it could be concluded that D-panthenol induces little effect on corneal epithelial regeneration, if any. It is not known whether this potential effect would vary according to dose or if a larger sample of patients was included to demonstrate this statistical difference.

## Figures and Tables

**Table 1:** Preoperative and operative data.

	Group A	Group B	*P* value
Preoperative sphere (D)	−2.9 ± 2 (−6.5–1.5)	−2.4 ± 2.6 (−6.25–4.0)	>0.05
Preoperative cylinder (D)	−0.68 ± 0.4 (−1.75–0)	−0.66 ± 0.47 (−1.5–0)	>0.05
Ablation zone (mm)	6.25 ± 0.35 (6–7)	6.25 ± 0.35 (6–7)	>0.05
Ablation depth (*µ*m)	51.5 ± 19.5 (19–90)	50.6 ± 20.7 (19–95)	>0.05

**Table 2:** First postoperative stage (epithelial defect).

		Day 1	Day 2	Day 3
UCVA (log MAR)	Group A	0.22 ± 0.18 (−0.1–0.5)	0.51 ± 0.31 (0.2–0.9)	0.51 ± 0.33 (0.1–1.0)
	Group B	0.24 ± 0.24 (−0.2–0.7)	0.52 ± 0.29 (0.2–1.0)	0.54 ± 0.28 (0.15–1.0)
	*P* value	>0.05	>0.05	>0.05

Healing (%)	Group A	52.8 ± 16.1 (30–80)	94.2 ± 12.2 (60–100)	99.1 ± 2.8 (90–100)
	Group B	50.8 ± 16.9 (15–80)	96.5 ± 4.4 (90–100)	99.1 ± 0.28 (99–100)
	*P* value	>0.05	>0.05	>0.05

Comfort	Group A	3.33 ± 1.4 (0–5)	1.71 ± 1.34 (0–4)	0.58 ± 0.9 (0–3)
	Group B	3.61 ± 1.09 (2–5)	1.63 ± 1.43 (0–4)	0.5 ± 1.0 (0–3)
	*P* value	>0.05	>0.05	>0.05

**Table 3:** Second postoperative stage (epithelial and stromal remodeling).

		Week 1	Week 2	Week 3	Month 1	Month 2
UCVA (log MAR)	Group A	0.1 ± 0.19 (−0.1–0.7)	0.63 ± 0.39 (0–1)	0.09 ± 0.1 (0–0.3)	0.07 ± 0.13 (−0.1–0.4)	0.03 ± 0.08 (−0.1–0.15)
	Group B	0.19 ± 0.19 (0–0.7)	0.32 ± 0.29 (0–0.8)	0.13 ± 0.11 (0–0.3)	0.11 ± 0.16 (−0.1–0.4)	0.02 ± 0.1 (−0.1–0.15)
	*P* value	<0.05^*∗*^	>0.05	>0.05	>0.05	>0.05

Residual cylinder (D)	Group A	0.42 ± 0.43 (0–1.25)	0.76 ± 0.54 (0–2.0)	0.59 ± 0.32 (0.25–1.0)	0.73 ± 0.5 (0–2.0)	0.62 ± 0.46 (0–1.5)
	Group B	0.7 ± 0.6 (0–1.75)	0.85 ± 0.77 (0–1.75)	0.87 ± 0.29 (0.5–1.25)	0.76 ± 0.52 (0–2.0)	0.56 ± 0.32 (0.25–1.25)
	*P* value	<0.05^*∗*^	>0.05	>0.05	>0.05	>0.05

Haze (no/epithelial/stromal) (%)	Group A	37.5/62.5/0	30/70/0	50/50/0	50/28.6/21.4	57.1/0/42.9
	Group B	6.3/93.8/0	20/80/0	50/50/0	42.9/21.4/35.7	71.4/0/28.6
	*P* value	>0.05	>0.05	>0.05	>0.05	>0.05

^*∗*^Significant.

## Data Availability

The data used to support the findings of this study are available from the corresponding author upon request.
